# Microstructure Evolution and Mechanical Properties of Dual-Phase AlCrFe_2_Ni_2_ High-Entropy Alloy Under High-Strain-Rate Compression

**DOI:** 10.3390/ma18061191

**Published:** 2025-03-07

**Authors:** Hang Yan, Yu Wang, Xilin Gan, Yong Dong, Shichao Liu, Shougang Duan, Lingbo Mao

**Affiliations:** 1 Innovation & Interdisciplinary Institute of Low Carbon Metallurgical Engineering, School of Materials and Energy, Guangdong University of Technology, Guangzhou 510006, China; yanhang0317@163.com (H.Y.); 17502008923@163.com (X.G.); sgduan0410@163.com (S.D.); lbmao@gdut.edu.cn (L.M.); 2 School of Materials Science and Engineering, North University of China, Taiyuan 030051, China; wangyu@nuc.edu.cn; 3 School of Iron and Steel, Soochow University, Suzhou 215021, China; sc_liu@suda.edu.cn; 4 School of Materials Science and Engineering, South China University of Technology, Guangzhou 510640, China

**Keywords:** high-entropy alloys, dynamic compression, adiabatic shear bands, microstructure

## Abstract

This paper investigates the effect of strain rate on the mechanical deformation and microstructural development of dual-phase AlCrFe_2_Ni_2_ high-entropy alloy during quasi-static and dynamic compression processes. It is revealed that the as-cast AlCrFe_2_Ni_2_ alloy is composed of a mixture of FCC, disordered BCC, and ordered B2 crystal structure phases. The alloy shows excellent compressive properties under quasi-static and dynamic deformation. The yield strength exceeds 600 MPa while the compressive strength is more than 3000 MPa at the compression rates of 30% under quasi-static conditions. Under dynamic compression conditions, the ultimate compression stresses are 1522 MPa, 1816 MPa, and 1925 MPa with compression strains about 12.8%, 14.7%, and 18.2% at strain rates of 1300 s^−1^, 1700 s^−1^ and 2100 s^−1^, respectively. The dynamic yield strength is approximately linear with strain rate within the specified range and exhibit great sensitivity. The strong localized deformation regions (i.e., adiabatic shear bands (ASBs)) appear in dynamically deformed samples by dynamic recrystallization due to the conflicting processes of strain rate hardening and heat softening.

## 1. Introduction

For conventional alloys, such as titanium alloys, magnesium alloys, and aluminum alloys, they are traditionally designed as one or two principal elements with minor additions of the other elements. Recently, a new alloy system, i.e., high-entropy alloys (HEAs), was designed differently from traditional counterparts. For HEAs, four or more principal elements are present in proportions of 5% to 35%, with the mixing entropy more than 1.5R [1,2]. HEAs usually form a simple solid solution phase [1], for instance, BCC, FCC, and HCP phases. These phases can either exist alone or as a dual-phase coexistence [2]. Based on previous research, single-phase HEAs with a BCC structure exhibit low plasticity properties but high tensile strength, whereas HEAs with a single-phase FCC structure typically have good plasticity but low tensile strength [3,4,5]. In order to balance the properties of HEAs, novel eutectic high-entropy alloys (EHEAs) consisting of a composite microstructure with FCC and BCC phases were designed and proposed by Lu et al. [6]. Until now, great potential has been recognized in many applications for these EHEAs, owing to their excellent fracture toughness [7], good cryogenic properties [7,8], exceptional wear, and fatigue resistances [9,10]. In addition, it is simple to adapt EHEAs with good flowability, castability, and high strength and ductility to large-scale industrial manufacturing [11,12].

Currently, several EHEA systems have been developed, including AlCoCrFeNi_2.1_ [6], Al_17_Ni_34_Ti_17_V_32_ [13], AlCr_1.3_TiNi_2_ [14], and AlCrCuFeNi_2_ [15]. Owing to better practicality and low cost, the AlCrFe_2_Ni_2_ alloy, with similar microstructure and properties to the AlCoCrFeNi_2.1_ alloy, was investigated. It is indicated that the AlCrFe_2_Ni_2_ alloy consists of the FCC and A2/B2 spinodal decomposition phase and presents better fracture strength and ductility [16]. In addition, the dynamic mechanical response of HEAs has also received a lot of attention. To use HEAs in extreme industrial environments, there is an urgent need for a comprehensive understanding of their performance and mechanisms during high-speed compression [17,18,19,20,21].

According to the available literature, in addition to the dislocation-related mechanism, twinning mechanism, grain boundary-related mechanism, diffusion creep mechanism, and dynamic recrystallization mechanism, the shear bands are formed in HEAs at high strain rates or under large deformation conditions [17,18,19,20,21]. The adiabatic shear band (ASB) is a localized region formed by a very large shear deformation in a very short time. In this region, along with the adiabatic temperature rise, the microstructure and properties of the material undergo significant changes, such as grain refinement and an increase in dislocation density. The ASB is an ideal mode of deformation, and the presence of the ASB is conducive to the enhancement of the penetration properties of the material. These traits differ significantly from those that occur when quasi-static characteristics are present. The dynamic behavior of Al_0.6_CoCrFeNi biphasic (FCC and BCC) alloys was examined by Wang et al. [22]. The findings demonstrated that the yield strength could be clearly increased by increasing the strain rate. Tian et al. found that the AlCoCrFeNi alloy exhibited extremely high strain rate sensitivity by investigating the alloy at different strain rates [23]. The dynamic deformation mechanism of the CrCoFeNi high-entropy alloy was investigated by Cao et al. [24]. It was found that massive dislocation nucleation and drag increased the strain rate sensitivity of the alloy at high strain rates, and the alloy also exhibited significant strain-hardening capability. Yuan et al. prepared Ni_32_Co_30_Cr_10_Fe_10_Al_18_ EHEAs by laser metal deposition, and it was found that the presence of the FCC phase caused the alloy to exhibit high ductility and strain-hardening ability at high temperatures and high strain rates [25]. Jeong Min Park et al. [26] found that the CoCrFeMnNi alloy showed strong strain rate sensitivity. Compression experiments on the AlCrCuFeNi_2_ alloy at a strain rate of 10^−3^ s^−1^~3000 s^−1^ were conducted by Ma et al. [15]. They discovered that over a range of strain rates there was a roughly linear connection between dynamic yield strength and strain rate. N. Kumar et al. [27] conducted compression tests on the Al_0.1_CrCoFeNi alloy using split-Hopkinson pressure bar (SHPB) and noted notable work hardening and strong strain rate sensitivity, as well as a large number of twinned grains [28].

Herein, in the present study, the primary emphasis is to provide more detailed insights into the compression behavior of the dual-phase AlCrFe_2_Ni_2_ alloy under both static compression and dynamic impact. In this context, the impact of strain rates on the microstructural development and mechanical characteristics of the AlCrFe_2_Ni_2_ alloy is assessed using the SHPB technique. Correspondingly, these fundamental processes for the development of microstructural features and mechanical characteristics are also examined and explained.

## 2. Experimental

### 2.1. Alloy Fabrication

For the present study, the experimental AlCrFe_2_Ni_2_ alloys were fabricated by using a vacuum high-frequency induction melting furnace, and all raw elemental metals have purity levels exceeding 99.9 wt.%. Melting and casting were conducted by backfilling with argon (Ar) under vacuum pressure up to 3 × 10^−3^ Pa at 0.01 atm.

### 2.2. Property Testing

For the static tests, cylindrical specimens that were about 10 mm tall and 5 mm in diameter were wire-cut and then ground. The deformation behavior of AlCrFe_2_Ni_2_ alloy at the strain rate in the range of 10^−4^ s^−1^~10^0^ s^−1^ was examined using a universal testing machine (AGS-X Plus100 KN, Japan), three samples being tested at each strain rate.

Following compression tests, samples were sliced along the stress loading direction, and silicon carbide paper with mechanical thinning was used to mechanically polish the surface parallel to the compression direction. EBSD analysis was used for microstructural characterization and orientation imaging microscope analysis. Samples measuring around 8 mm in diameter and 5 mm in height were prepared for dynamic compression. An SHPB with strain rates ranging from 1300 s^−1^ to 2100 s^−1^ was used for dynamic compression experiments at ambient temperature. The force applied to the deformation zone when the cylindrical sample is dynamically loaded by the SHPB was computed using the information gathered by the strain gauges on the incident and transmitted bars. The function of time t was used to calculate the strain rate $\overset{•}{\varepsilon }$, true strain ε and true stress σ. The following is how the equations are expressed: [29]:

(1) $$\overset{•}{\varepsilon }=-\frac{2C_{0}}{L_{0}}\varepsilon_{r}\left(t\right)$$

(2) $$\varepsilon =-\frac{2C_{0}}{L_{0}}\int_{0}^{l}\varepsilon_{r}\left(t\right)dt$$

(3) $$\sigma =\frac{A_{0}}{A}Ε\varepsilon_{t}\left(t\right)$$

where Ε is the elastic modulus of the incident and transmission bars, and $A_{0}$. And $C_{0}$ are the wave propagation velocities in the bars. A is the cross-section area of the incident and transmission bars, and $L_{0}$ is the height and cross-section area of sample. $\varepsilon_{r}\left(t\right)$ and $\varepsilon_{t}\left(t\right)$ are the experimentally measured strains of the incident and transmitted stress pulses on the SHPB. You can find more information about the SHPB and the stress and strain calculations elsewhere.

### 2.3. Microstructure Characterization

An X-ray diffractometer (XRD, EMPYREAN with Cu radiation target) was used to identify the phase. It has a scanning rate of 4° min^−1^ and a 2θ scanning range of 20° to 100°. Energy dispersive spectrometry (EDS) in conjunction with scanning electron microscopy (SEM, JEOL-JSM-IT800) and transmission electron microscopy (TEM, Talos F200S) were utilized to describe the microstructural evolution and deformation process of the AlCrFe_2_Ni_2_ alloy. Metallographic polishing and twin-jet electropolishing (electrolyte: 90 vol.% ethanol + 10 vol.% perchloric acid) were used to produce the thin-foil sample for TEM analysis. Samples were prepared by mechanically thinning after normal metallographic polishing for electron backscatter diffraction (EBSD) examination.

## 3. Results and Discussion

### 3.1. Microstructural Analysis

**Figure 1:**
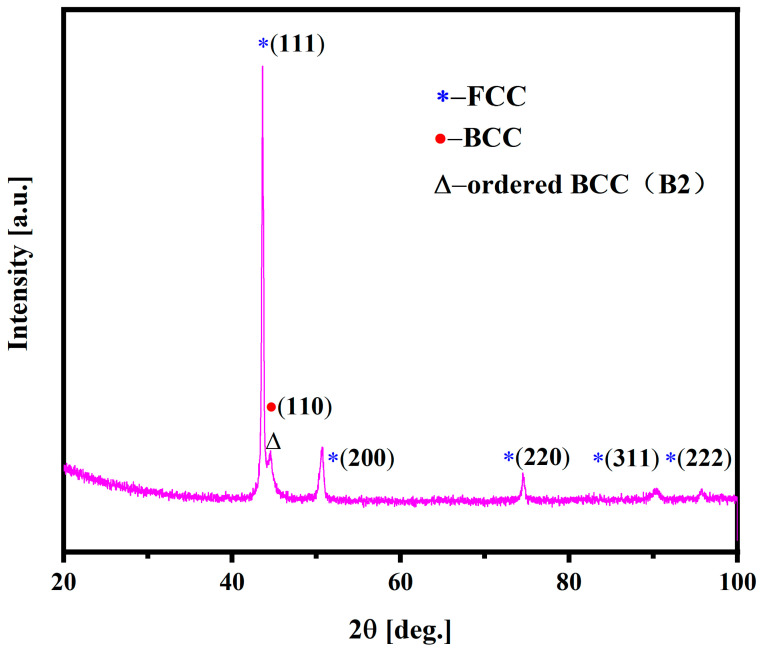
X-ray diffraction of the as-cast AlCrFe_2_Ni_2_ alloy.

Figure 1 shows the X-ray diffraction pattern of the as-cast AlCrFe_2_Ni_2_ alloy. The XRD result indicates that the alloy is composed of a mixture of FCC, disordered BCC, and ordered B2 crystal structures. In addition, due to the same crystal structure and similar lattice parameter, the pattern presents the overlapping disordered BCC and B2 Bragg reflection, which was also found in the other HEAs [30].

**Figure 2:**
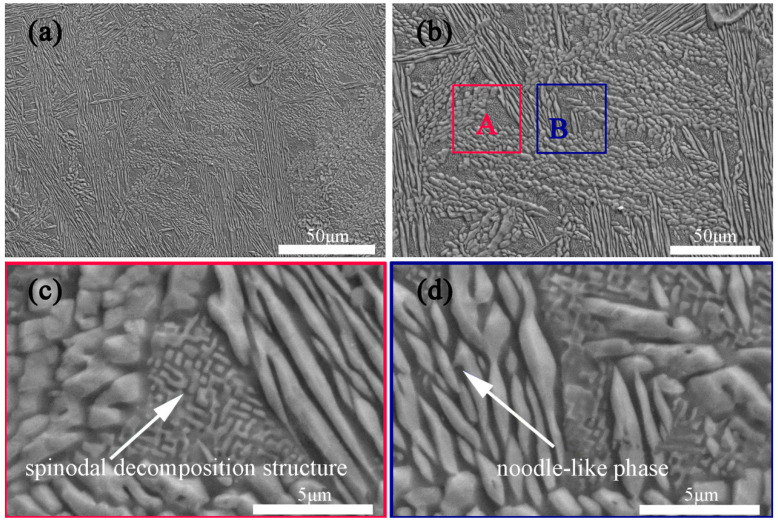
Microstructure of the as-cast AlCrFe_2_Ni_2_ alloy: (**a**) SEM image; (**b**) the high-magnified SEM secondary electron image; (**c**) and (**d**) are magnified images of regions A and B in (**b**), respectively.

Figure 2 shows the SEM images of the as-cast AlCrFe_2_Ni_2_ alloy. From Figure 2a, it can be seen that the alloy mainly consists of noodle-like phases and triangular phases. Further observed by the high-magnified SEM image in Figure 2b, the length and size of the noodle-like phases are not uniform, the other phases are not always triangular in shape, and there are various other shapes. Figure 2c,d shows enlarged images of Figure 2b. The area of the triangle that can be seen in Figure 2c is composed of interconnected light and dark microstructures, which is characteristic of the microstructure of the spinodal decomposition microstructure. The noodle-like phases are clearly visible in Figure 2d, and there are fine dark phases present between the noodle-like phases [16,31,32].

**Figure 3:**
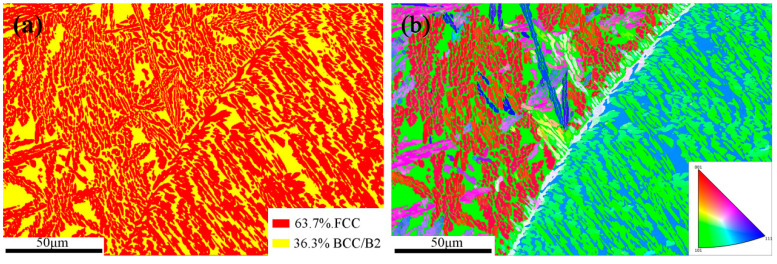
(**a**) EBSD phase diagram of the as-cast AlCrFe_2_Ni_2_ alloy; (**b**) EBSD inverse pole figure (IPF).

Figure 3 shows the EBSD phase diagram and IPF diagram of the as-cast AlCrFe_2_Ni_2_ alloy. From Figure 3a, it can be seen that the alloy is mainly formed by the FCC phase and BCC/B2 phase, which is consistent with the XRD analysis results, where the FCC phase accounts for 63.7% and the BCC phase accounts for 36.3%. Combined with Figure 2, the noodle-like phase is the FCC phase, and the other phases are the BCC and B2 phases. As can be seen from Figure 3b, the grain orientations of the noodle-like phases are disordered, whereas the orientations of the alternating phases are partly uniform and partly disordered, which suggests that the remaining phases are composed of the BCC phase and the B2 phase.

**Figure 4:**
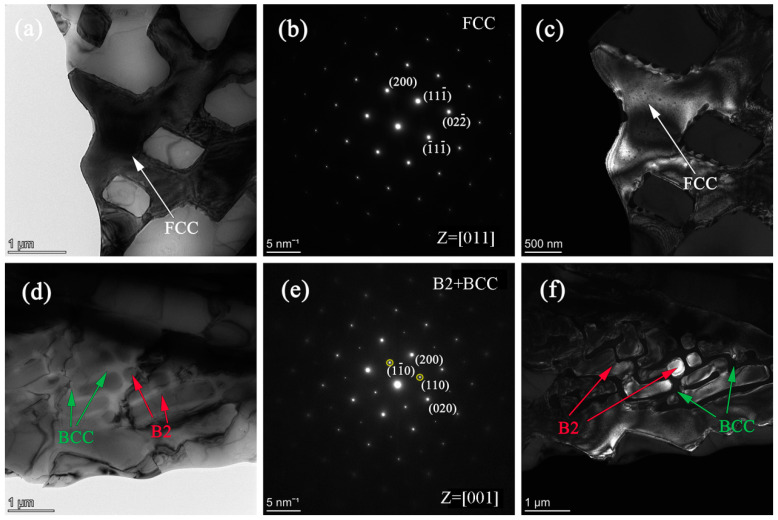
(**a**,**d**) Bright-field images of the AlCrFe_2_Ni_2_; (**b**,**e**) selected area diffractions from zone axes [011] and [001], respectively; (**c**,**f**) dark-field images.

**Figure 5:**
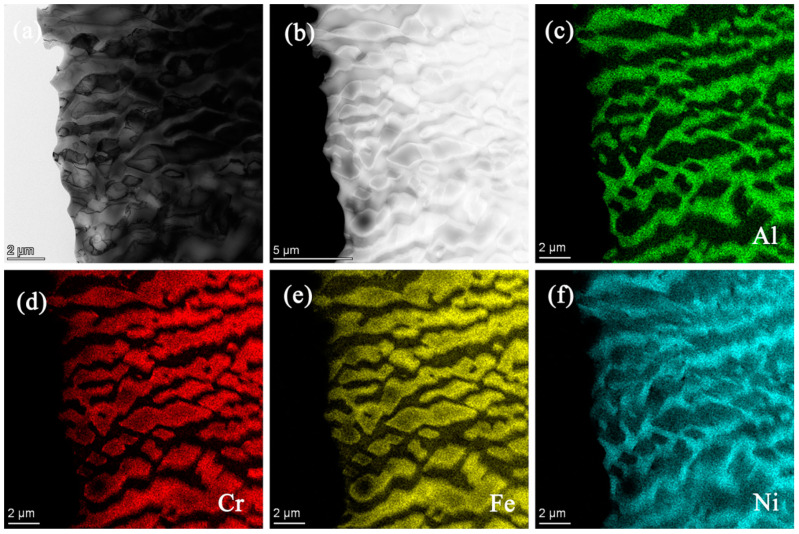
TEM images of the as-cast AlCrFe_2_Ni_2_ alloy: (**a**) bright-field TEM image; (**b**–**f**) the element distribution by TEM-EDS.

Further detailed analysis on the microstructure of the AlCrFe_2_Ni_2_ alloy was carried out by TEM, as seen in Figure 4. Figure 4a,c shows bright-field images of the AlCrFe_2_Ni_2_ alloy. It is evident that the alloy is composed of noodle-like phases and a spinodal decomposition microstructure, which is an alternating mixture of the two phases. Figure 4b,d shows the selected area electron diffraction (SAED) pattern of the alloy AlCrFe_2_Ni_2._ From Figure 4b and the dark-field image (Figure 4c), it can be obtained that the noodle-like phase is the FCC phases, which is also in agreement with the results of the EBSD phase map. From Figure 4d, it can be seen that the modulated structure is a mixture consisting of BCC and B2 phases, and combined with the dark-field image (Figure 4f), in the modulated structure, the matrix has a BCC structure and the particle-shaped phase has a B2 structure. Figure 5b–f shows the element distribution of the AlCrFe_2_Ni_2_ alloy, from which it can be seen that the AlCrFe_2_Ni_2_ alloy forms BCC (Cr, Fe)-rich inter-dendritic matrices, and B2 (Al, Ni)-rich particles (similar to those in Ref. [33]).

### 3.2. Mechanical Characteristics Under Compression Testing

**Figure 6:**
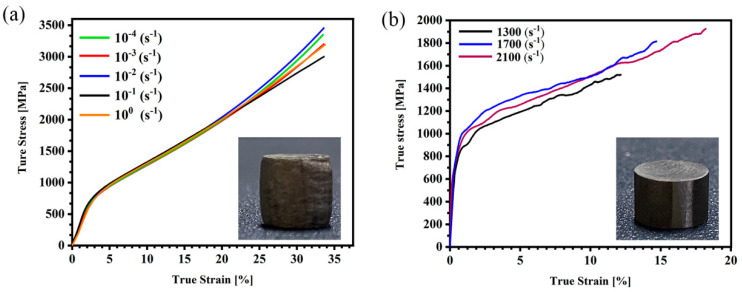
(**a**) Static compression stress–strain curves of the AlCrFe_2_Ni_2_ high-entropy alloy under quasi-statics; (**b**) dynamic compression stress–strain curves at various strain rates.

Figure 6 shows the true stress–strain curve of the AlCrFe_2_Ni_2_ alloy at different strain rates. The true compression stress–strain curves of the AlCrFe_2_Ni_2_ alloy under quasi-static deformation at room temperature are shown in Figure 6a. From Figure 6a, it can be seen that when the strain rate is 10^−4^ s^−1^–10^0^ s^−1^, the alloy shows significant work hardening, but there is no significant change in the yield strength value with increasing strain rate. The inset in Figure 6a shows the sample after compression, and the sample did not fracture, which indicates that the AlCrFe_2_Ni_2_ alloy has good plasticity. Thus, the maximum strain is taken to be 30%, and the corresponding ultimate compression stress are more than 3000 MPa. Correspondingly, the compression properties of the AlCrFe_2_Ni_2_ alloy are shown in Table 1. Figure 6b presents the compression performance of the AlCrFe_2_Ni_2_ alloy at high strain rates. It can be seen from Figure 6b that the yield strength changes significantly with the increase in strain rate. Furthermore, a positive strain rate effect is indicated by the dynamic yield stress, and the flow stress shows an upward trend with the rise of strain rate. The compression properties of the AlCrFe_2_Ni_2_ alloy are shown in Table 2. In conjunction with the accompanying graph from Figure 6b, because of the transient loading time, the surface of the AlCrFe_2_Ni_2_ alloy has not changed in any way, the alloy has failed, and therefore severe changes should have occurred within the alloy at high strain rates [34].

The strain rate sensitivity (m) is used to describe the dependence of strength/stress on strain rate. It can be defined by the slope of the log strength σ versus log strain rate $\overset{•}{\varepsilon }$ at constant strain ∂:

(4) $$m=\frac{\partial \ln\sigma }{\partial \ln\overset{•}{\varepsilon }}$$

The yield strengths are roughly 606 MPa and 658 MPa at strain rates of 10^−4^ s^−1^ and 10^0^ s^−1^, respectively. The AlCrFe_2_Ni_2_ alloy m under quasi-static deformation is estimated to be around 0.0089. It is worth noting that it is of the same order of magnitude as the conventional metals: Cu (~0.006) and Ni (~0.0028) [34], but an order of magnitude lower than that of partially eutectic high-entropy alloys (~0.028) [35]. The yield strengths are roughly 892 MPa and 1132 MPa at strain rates of 1300 s^−1^ and 2100 s^−1^, respectively, and the m of the AlCrFe_2_Ni_2_ alloy is estimated to be about 0.42 at high strain rates, which is much higher than the value under quasi-static deformation, and is also consistent with the image pattern of the true stress–strain curve. The present reason for the sudden increase in the strain rate sensitivity coefficient may be due to phonon drag effects [24,36]. Elastic lattice vibrations that go through crystals are called phonons. The phonon drag effect is the result of the interaction between phonons and dislocations, which creates viscous resistance to dislocation sliding. The phonon drag effect is known to be caused by a number of ways [18].

**Figure 7:**
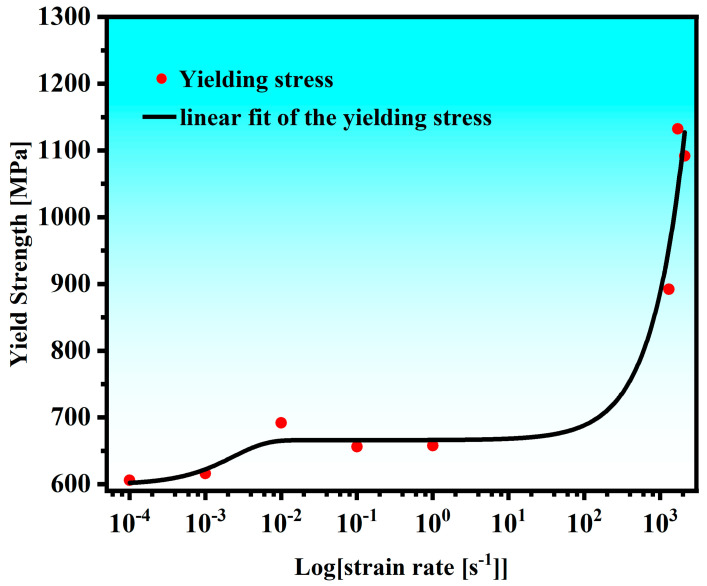
The yield strength as a function of the logarithmic strain rate for the AlCrFe_2_Ni_2_ high-entropy alloy.

The yield strength for the AlCrFe_2_Ni_2_ alloy is plotted against the logarithmic strain rate in Figure 7. It can be seen from Figure 7 that the trend of yield strength tends to a straight line when the strain rate is 10^−4^ s^−1^–10^3^ s^−1^, and when the strain rate is greater than 10^3^ s^−1^, the yield strength of the alloy has a clear upward trend with the increase in strain rate. It is evident that under dynamic conditions, the effect of strain rate on yield strength is significantly higher. They are therefore divided into two regions where the strength fluctuates with the strain rate: the area where phonon drag influences dislocation motions ($\overset{˙}{\varepsilon }$ > 10^3^ s^−1^) and the area of thermally induced dislocation glide ($\overset{˙}{\varepsilon }$ < 10^3^ s^−1^) [20,27,37,38]. In the region of $\overset{˙}{\varepsilon }$ < 10^3^ s^−1^, the dislocations engage with the surrounding regions’ isotropic phonon flux, and the dislocation velocity is so low that the phonon drag effect barely affects the dislocation motion [18,27]. In the region of $\overset{˙}{\varepsilon }$ >10^3^ s^−1^, viscous phonon drag significantly affects dislocation motion [18,27,39]. Fast moving dislocations would cause phonon scattering through the strain field. Moreover, dislocations could absorb phonon energy, which leads to phonon scattering, and the kinematic viscosity of dislocations increases due to the phonon dispersion.

### 3.3. Microstructure Evolution

**Figure 8:**
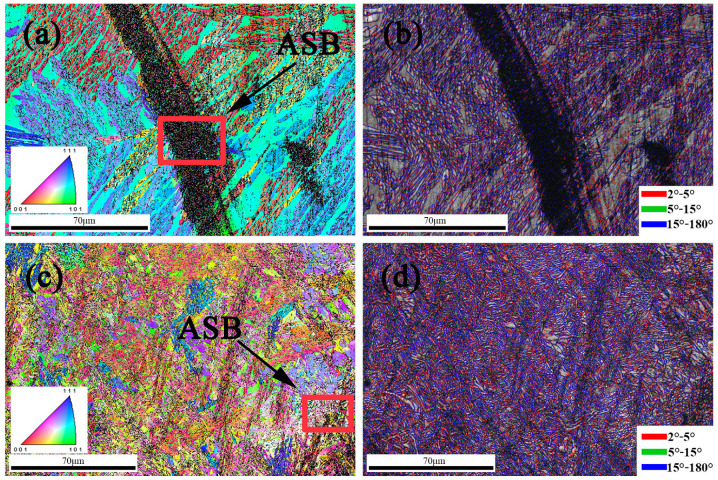
Orientation image maps with the IPF and IQ + misorientation boundary maps of the samples after high-strain compression: (**a**,**b**) at 1300 s^−1^; (**c**,**d**) at 2100 s^−1^.

The IPF and IQ + misorientation border maps (BMs) at a strain rate of 1300 s^−1^ and 2100 s^−1^ are displayed in Figure 8, respectively. From Figure 8a, it can be seen that there is a distinct black area in the center, which is an ASB region, while in Figure 8c, only smaller discontinuous ASB zones can be observed. In addition, a large number of highly refined submicron grains can be seen in the red-boxed portion of Figure 8a,c. In Figure 8c, although many ASB regions are observed, the number of fine grains with HAGBs is significantly less than that in the regions with a strain rate of 1300 s^−1^. The dynamic recrystallization (DRX) is the cause of the grain refinement observed in the dynamically deformed materials, such as the ASBs frequently seen in a variety of deformation materials, which may be the source of DRX [38,40,41]. The ASB at 1300^−1^ s^−1^ is a narrow region of high-concentration fine grains. These ASBs cause cracks to form, and eventually shear damage, which explains the poor performance of strain rate at 1300^−1^ s^−1^. The emergence of ASBs may be due to the conflicting processes of strain rate hardening and heat softening [18]. These two processes, strain-hardening and heat-softening effects brought on by dislocation proliferation/stacking or twin evolution, would compete with one another during dynamic deformation. The strain-hardening effect would eventually be overcome by the contribution of thermal softening to the flow stresses, which would result in the production of ASBs. As can be seen from Figure 8b,d, there are a large number of blue regions at high strain rates. The blue region indicates high-angle grain boundaries (HAGBs) with dislocation angles more than 15°, whereas the red and green lines correspond to low-angle grain boundaries (LAGBs) less than 15°. LAGBs provide favorable conditions for dislocation proliferation due to their special structure and higher energy states, which explains the increase in yield strength at high strain rates.

**Figure 9:**
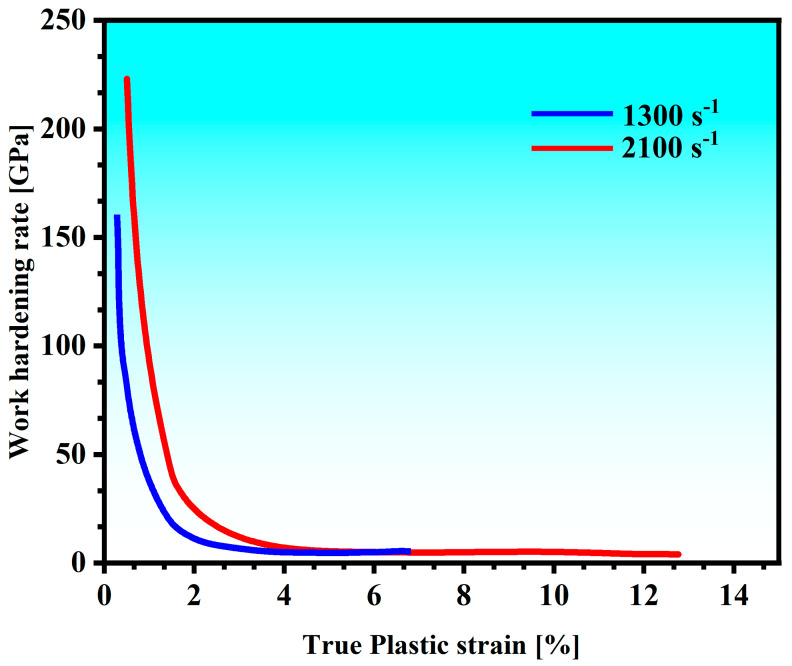
The work-hardening rate (WHR): dσ/dε (σ is the flow stress and ε is the plastic strain).

The work-hardening rate (WHR, dσ/dε, where σ is the flow stress and ε is the plastic strain) behavior as a function of the plastic strain at a strain rate of 1300 s^−1^ and 2100 s^−1^ is displayed in Figure 9. It has been recognized that higher strain rates produce a larger WHR, especially in the initial plasticity stage. This characterization can be explained by the expression [42]:

(5) $$\bar{\nu }=A\tau^{m}$$

where A and m are the constants for specific materials and $\bar{\nu }$ is the dislocation movement velocity. The initial shear stress for dislocation movement is denoted by τ. The WHR and the strain rates are positively correlated, as seen in Figure 9. The higher strain rates result in the larger shear stress and the higher dislocation motion rate. Two distinct zones can be seen inside the curve. In one region (true plastic strain < 2%), the strain-hardening rate (SHR) drops significantly as the strain increases. In this region, the strain rate is proportional to the work-hardening rate, which is related to the formation of adiabatic shear bands. At a strain rate of 2100 s^−1^, although there is also the formation of ASBs, the distribution of the shear bands of the fine scattering does not cause the alloy to fail quickly, but rather has a certain strengthening effect. In another region (true plastic strain > 2%), the strain-hardening rate drops less steeply; the plastic deformation in this region is dominated by dislocation slip and the rising dislocation annihilation rate, but the SHR does not rise due to the effect of thermal softening [43].

## 4. Conclusions

In this work, the effect of strain rate on the microstructural evolution and compression behavior of the dual-phase AlCrFe_2_Ni_2_ alloy was studied. The main conclusions are as follows:

1. The as-cast AlCrFe_2_Ni_2_ alloy is composed of a mixture of Fe,Ni-enriched FCC phases, disordered Fe,Cr-enriched BCC phases, and ordered Al,Ni-enriched B2 phases.
2. The AlCrFe_2_Ni_2_ alloy shows excellent compressive properties under quasi-static deformation. The yield strength exceeds 600 MPa while the compressive strength is more than 3000 MPa at the compression rates of 30%.
3. The ultimate compression stresses are 1522 MPa, 1816 MPa, and 1925 MPa with compression strains about 12.8%, 14.7%, and 18.2% at strain rates of 1300 s^−1^, 1700 s^−1^, and 2100 s^−1^, respectively. Strain rate sensitivity ranges from 0.0089 at low strain (10^−4^ s^−1^–10^0^ s^−1^) to 0.42 at high strain rates (1300 s^−1^–2100 s^−1^), which is due to the phonon drag effect at high strains.
4. At high-strain-rate compression, a adiabatic shear phenomenon occurs. The adiabatic shear bands formed by dynamic recrystallization due to the conflicting processes of strain rate hardening and heat softening.

## Figures and Tables

**Table 1:** A summary of the compressive properties of the AlCrFe_2_Ni_2_ alloy under quasi-static deformation at room temperature.

Strain Rate [s^−1^]	Yield Strength [MPa]	Plastic Strain [%]
1 × 10^−4^	606.1 ± 3.0	>30.0
1 × 10^−3^	616.3 ± 3.1	>30.0
1 × 10^−2^	691.6 ± 3.5	>30.0
1 × 10^−1^	655.8 ± 3.3	>30.0
1 × 10^0^	658.3 ± 3.3	>30.0

**Table 2:** A summary of the compressive properties of the AlCrFe_2_Ni_2_ alloy under dynamic deformation at room temperature.

Strain Rate [s^−1^]	Yield Strength [MPa]	Compressive Stress [MPa]	Plastic Strain [%]
1300	891.9 ± 4.5	1521.8 ± 7.6	12.8 ± 2.3
1700	1132.4 ± 5.7	1815.5 ± 9.1	14.7 ± 2.0
2100	1091.7 ± 5.5	1925.3 ± 9.6	18.2 ± 1.6

## Data Availability

The original contributions presented in this study are included in the article. Further inquiries can be directed to the corresponding author.

## References

[1] Yeh J.W., Chen S.K., Lin S.J., Gan J.Y., Chin T.S., Shun T.T., Tsau C.H., Chang S.Y. (2004). Nanostructured High-Entropy Alloys with Multiple Elements: Novel Alloy Design Concepts and Outcomes. Adv. Eng. Mater..

[2] Li W.D., Liaw P.K., Gao Y. (2018). Fracture resistance of high entropy alloys: A review. Intermetallics.

[3] Liu W.H., He J.Y., Huang H.L., Wang H., Lu Z.P., Liu C.T. (2015). Effects of Nb additions on the microstructure and mechanical property of CoCrFeNi high-entropy alloys. Intermetallics.

[4] He J.Y., Liu W.H., Wang H., Wu Y., Liu X.J., Nieh T.G., Lu Z.P. (2014). Effects of Al addition on structural evolution and tensile properties of the FeCoNiCrMn high-entropy alloy system. Acta Mater..

[5] Senkov O.N., Wilks G.B., Scott J.M., Miracle D.B. (2011). Mechanical properties of NbMoTaW and VNbMoTaW refractory high entropy alloys. Intermetallics.

[6] Lu Y.P., Dong Y., Guo S., Jiang L., Kang H.J., Wang T.M., Wen B., Wang Z.J., Jie J.C., Ruan H.H. (2014). A Promising New Class of High-Temperature Alloys: Eutectic High-Entropy Alloys. Sci. Rep..

[7] Gludovatz B., Hohenwarter A., Catoor D., Chang E.H., George E.P., Ritchie R.O. (2014). A fracture-resistant high-entropy alloy for cryogenic applications. Science.

[8] Otto F., Dlouh A., Somsen C.H., Bei H., Eggeler G., George E.P. (2013). The influences of temperature and microstructure on the tensile properties of a CoCrFeMnNi high-entropy alloy. Acta Mater..

[9] Chuang M.H., Tsai M.H., Wang W.R., Lin S.J., Yeh J.W. (2011). Microstructure and wear behavior of Al_x_Co_1.5_CrFeNi_1.5_Tiy high-entropy alloys. Acta Mater..

[10] Hemphill M.A., Yuan T., Wang G.Y., Yeh J.W., Tsai C.W., Chuang A., Liaw P.K. (2012). Fatigue behavior of Al_0.5_CoCrCuFeNi high entropy alloys. Acta Mater..

[11] Lu Y.P., Dong Y., Jiang H., Wang Z.J., Cao Z.Q., Guo S., Wang T.M., Li T.J., Liaw P.K. (2020). Promising properties and future trend of eutectic high entropy alloys. Scr. Mater..

[12] Lu Y.P., Gao X.Z., Jiang L., Chen Z.N., Wang T.M., Jie J.C., Kang H.J., Zhang Y.B., Guo S., Ruan H.H. (2017). Directly cast bulk eutectic and near-eutectic high entropy alloys with balanced strength and ductility in a wide temperature range. Acta Mater..

[13] Wang M.L., Lu Y.P., Lan J.G., Wang T.M., Zhang C., Cao Z.Q., Li T.J., Liaw P.K. (2023). Lightweight, ultrastrong and high thermal-stable eutectic high-entropy alloys for elevated-temperature applications. Acta Mater..

[14] Wang M.L., Lu Y.P., Lan J.G., Wang T.M., Zhang C.A., Cao Z.Q., Li T.J., Liaw P.K. (2021). A novel bulk eutectic high-entrogy alloy with outstanding as-cast specific yield strengths at elevated temperatures. Scr. Mater..

[15] Ma S.G., Jiao Z.M., Qiao J.W., Yang H.J., Zhang Y., Wang Z.H. (2016). Strain rate effects on the dynamic mechanical properties of the AlCrCuFeNi2 high-entropy alloy. Mater. Sci. Eng. A.

[16] Dong Y., Gao X.X., Lu Y.P., Wang T.M., Li T.J. (2016). A multi-component AlCrFe_2_Ni_2_ alloy with excellent mechanical properties. Mater. Lett..

[17] Li Z., Zhao S., Diao H., Liaw P.K., Meyers M.A. (2017). High-velocity deformation of Al_0.3_CoCrFeNi high-entropy alloy: Remarkable resistance to shear failure. Sci. Rep..

[18] Meyers M.A. (1994). Dynamic Behavior of Materials.

[19] Cho K.M., Lee S.H., Nutt S.R., Duffy J. (1993). Adiabatic shear band formation during dynamic torsional deformation of an HY-100 steel. Acta Mater..

[20] Lee H.S., Sohn S.S., Jeon C.W., Jo I.G., Lee S.K., Lee S.H. (2017). Dynamic compressive deformation behavior of SiC-particulate-reinforced A356 Al alloy matrix composites fabricated by liquid pressing process. Mater. Sci. Eng. A.

[21] Dirras G., Couque H., Lilensten L., Heczel A., Tingaud D., Couzinié J.-P., Perriére L., Gubicza J., Guillot I. (2016). Mechanical behavior and microstructure of Ti20Hf20Zr20Ta20Nb20 high-entropy alloy loaded under quasi-static and dynamic compression conditions. Mater. Charact..

[22] Wang L., Qiao J.W., Ma S.G., Jiao Z.M., Zhang T.W., Chen G., Zhao D., Zhang Y., Wang Z.H. (2018). Mechanical response and deformation behavior of Al_0.6_CoCrFeNi high-entropy alloys upon dynamic loading. Mater. Sci. Eng..

[23] Tian L., Jiao Z.M., Yuan G.Z., Ma S.G., Wang Z.H., Yang H.J., Zhang Y., Qiao J.W. (2016). Effect of Strain Rate on Deformation Behavior of AlCoCrFeNi High-Entropy Alloy by Nanoindentation. Mater. Eng. Perform..

[24] Cao T.Q., Zhang Q., Wang L., Wang L., Xiao Y., Yao J.H., Liu H.Y., Ren Y., Liang J., Xue Y.F. (2023). Dynamic deformation behaviors and mechanisms of CoCrFeNi high-entropy alloys. Acta Mater..

[25] Yuan K.B., Yao X.H., Yu Y.Q., Wang R.F., Chai Z.S., Zhou K.X., Wang Z.J. (2023). Dynamic thermomechanical response and constitutive modeling of eutectic high-entropy alloy. Int. J. Mech. Sci..

[26] Park J.M., Moon J., Bea J.W., Jang M.J., Park J., Lee S., Kim H.S. (2018). Strain rate effects of dynamic compressive deformation on mechanical properties and microstructure of CoCrFeMnNi high-entropy alloy. Mater. Sci. Eng..

[27] Kumar N., Ying Q., Nie X., Mishra R.S., Tang Z., Liaw P.K., Brenan R.E., Doherty K.J., Cho K.C. (2015). High strain-rate compressive deformation behavior of the Al_0.1_CrFeCoNi high entropy alloy. Mater. Des..

[28] Zhang Y., Zuo T.T., Tang Z., Gao M.C., Dahmen K.A., Liaw P.K., Lu Z.P. (2014). Microstructures and properties of high-entropy alloys. Prog. Mater. Sci..

[29] Wang B.F., Fu A., Huang X.X., Liu B., Liu Y., Li Z.Z., Zan X. (2016). Mechanical properties and microstructure of the CoCrFeMnNi high entropy alloy under high strain rate compression. Mater. Eng. Perform..

[30] Dong Y., Zhou K.Y., Lu Y.P., Gao X.X., Wang T.M., Li T.J. (2014). Effect of vanadium addition on the microstructure and properties of AlCoCrFeNi high entropy alloy. Mater. Des..

[31] Singh S., Wanderka N., Murty B.S., Glatzel U., Banhart J. (2011). Decomposition in multi-component AlCoCrCuFeNi high-entropy alloy. Acta Mater..

[32] Santodonato L.J., Zhang Y., Feygenson M., Parish C.M., Gao M.C., Weber R.J.K., Neuefeind J.C., Tang Z., Liaw P.K. (2015). Deviation from high-entropy configurations in the atomic distributions of a multi-principal-element alloy. Nat. Commun..

[33] Linden Y., Pinkas M., Munitz A., Meshi L. (2017). Long-period antiphase domains and short-range order in a B2 matrix of the AlCoCrFeNi high-entropy alloy. Scr. Mater..

[34] Moon J., Hong S.I., Bae J.W., Jang M.J., Yim D., Kim H.S. (2017). On the strain rate-dependent deformation mechanism of CoCrFeMnNi high-entropy alloy at liquid nitrogen temperature. Mater. Res. Lett..

[35] Tang Y., Wang R.X., Xiao B., Zhang Z.R., Li S., Qiao J.W., Bai S.X., Zhang Y., Liaw P.K. (2023). A review on dynamic-mechanical behaviors of high entropy alloys. Prog. Mater. Sci..

[36] Hong S.I., Moon J., Hong S.K., Kim H.S. (2017). Thermally activated deformation and the rate controlling mechanism in CoCrFeMnNi high entropy alloy. Mater. Sci. Eng. A.

[37] Hines J.A., Vecchio K.S. (1997). Recrystallization kinetics within adiabatic shear bands. Acta Mater..

[38] Chen W., Song B. (2011). Split Hopkinson (Kolsky) Bar.

[39] Conque H. (2014). The use of the direct impact Hopkinson pressure bar technique to describe thermally activated and viscous regimes of metallic materials. Philos. Trans. A Math. Phys. Eng. Sci..

[40] Meyers M.A., Nesterenko V.F., Lasalvis J.C., Xue Q. (2001). Shear localization in dynamic deformation of materials: Microstructural evolution and self-organization. Mater. Sci. Eng. A.

[41] Ahn D.H., Lee D.J., Kang M.J., Park L.J., Lee S.H., Kim H.S. (2016). Bi-modal structure of copper via room-temperature partial recrystallization after cryogenic dynamic compression. Metall. Mater. Trans. A.

[42] Bai J., Kou H.C., Wang J., Li J.S., Hu R. (2014). Strain rate response of a Ti-based metallic glass composite at cryogenic temperature. Mater. Lett..

[43] Jiang K., Ren T.F., Shan G.B., Ye T., Chen L.Y., Wang C.X., Zhao F., Li J.G., Suo T. (2020). Dynamic mechanical responses of the Al_0.1_CoCrFeNi high entropy alloy at cryogenic temperature. Mater. Sci. Eng. A.

